# Cost-effectiveness of rapid diagnostic tests, compared to microscopic tests, for the diagnosis and treatment of gestational malaria in Colombia from an institutional perspective

**DOI:** 10.1186/s12936-020-03472-6

**Published:** 2020-11-10

**Authors:** Deisy Cristina Restrepo-Posada, Jaime Carmona-Fonseca, Jaiberth Antonio Cardona-Arias

**Affiliations:** 1 University of Antioquia, Medellin, Colombia; 2 Department of Medicine, University of Antioquia, Medellin, Colombia; 3 School of Microbiology, University of Antioquia, Calle 70 Number 52–51, Block 5, office 103, Medellin, Colombia

**Keywords:** Cost-effectiveness assessment, Gestational malaria, Rapid diagnostic tests, Thick blood smear tests, Microscopic tests, Colombia

## Abstract

**Background:**

Gestational malaria is associated with negative outcomes in maternal and gestational health; timely diagnosis is crucial to avoid complications. However, the limited infrastructure, equipment, test reagents, and trained staff make it difficult to use thick blood smear tests in rural areas, where rapid testing could be a viable alternative. The purpose of this study was to estimate the cost-effectiveness of rapid tests type III (*Plasmodium falciparum*/*Plasmodium* spp P.f/pan) *versus* microscopic tests for the diagnosis and treatment of gestational malaria in Colombia.

**Methods:**

Cost-effectiveness analyses of gestational malaria diagnosis from an institutional perspective using a decision tree. Standard costing was performed for the identification, measurement and assessment phases, with data from Colombian tariff manuals. The data was collected from Health Situation Analysis, SIVIGILA and meta-analysis. Average and incremental cost-effectiveness ratio were estimated. The uncertainty was assessed through probabilistic sensitivity analysis.

**Results:**

The cost of rapid diagnostic tests in 3,000 pregnant women with malaria was US$66,936 and 1,182 disability adjusted life years (DALYs) were estimated. The cost using thick blood smear tests was US$50,838 and 1,023 DALYs, for an incremental cost-effectiveness of US$ 101.2. The probabilistic sensitivity analysis of rapid diagnostic tests determined that they are highly cost-effective in 70% of the cases, even below the US$1,200 threshold; also, they showed an incremental net monetary benefit of $150,000 when payer’s willingness is US$1,000.

**Conclusion:**

The use of rapid diagnostic tests for timely diagnosis and treatment of gestational malaria is a highly cost-effective strategy in Colombia, with uncertainty analyses supporting the robustness of this conclusion and the increased net monetary benefit that the health system would obtain. This strategy may help in preventing the negative effects on maternal health and the neonate at a low cost.

## Background

Malaria is a public health problem worldwide. In 2018, the World Health Organization (WHO) reported 228 million cases of the disease, more than 400,000 deaths, and Africa as the most affected region. In America, 21 countries are endemic with Brazil, Colombia, Guyana, Haiti, Peru, and Venezuela reporting more than 90% of cases [1, 2]. Morbidity and mortality rates are the highest in pregnant women, their fetuses, and children under 5 years of age, constituting the most vulnerable population groups. It is estimated that more than 125 million pregnant women are at risk of acquiring the infection; per year, pregnancy-associated malaria (PAM), gestational, placental and congenital malaria, causes 35% of low birth weight (LBW) cases, 26% of the cases of severe anaemia during pregnancy, 17.6% of maternal deaths, and 70% of the cases of intrauterine growth restriction [3]. Other conditions, such as cerebral malaria, severe malaria, pre-term birth, abortion, increased risk of co-infection and malnutrition, and intrauterine, neonatal, and infant mortality are also related to the disease [3–8].

This situation, together with the high burden of PAM has led to increased research efforts aimed at reducing its negative effects, mainly in high-risk groups. Works published include meta-analyses showing the effectiveness of intermittent preventive treatment in pregnant women (IPTp) in reducing the risk of maternal anaemia up to 40%, 39% of miscarriages and 19% of LBW, as well as the effectiveness of insecticide-treated bed nets, preventive treatment in HIV-positive pregnant women, and infant prophylaxis [9]. Likewise, systematizations on the burden of PAM, its effects on maternal and child health [10], and the efficacy of prophylactics [11–13] have also been published. This type of scientific evidence has allowed the WHO to implement strategies to reduce the incidence of the disease in highly endemic countries. However, such measures have not been enough to reach the targets in terms of reduction and elimination; even in America, both incidence and deaths have increased [1].

Given that the disease mainly affects poor countries, experts have recommended the inclusion of economic studies as a research priority, aimed at identifying effective, safe and cost-effective strategies that guarantee their provision by the government [14]. In response, several economic assessments have demonstrated the cost-effectiveness of PAM prevention [15–17]. In particular, a systematic review of economy studies on PAM, which implemented 9 search strategies in PubMed, Science Direct, Scielo, Google Scholar and Malaria in Pregnancy (MiP) Library, identified 22 studies [18] with the following results: most of the studies were mainly conducted in Africa and that did not include incremental or sensitivity analyses, reducing thus the internal and external validity of the conclusions; 90% were cost-effectiveness studies (in this type of economic study 82% evaluated IPTp) and the most common outcomes were LBW (65%), disability adjusted life years (DALYs) (55%), maternal anaemia (41%), and maternal malaria (29%).

These findings show that economic evaluations are still limited, demonstrating a gap in the knowledge about the economic impact of PAM, that the evidence is concentrated in Africa, where the WHO recommends the use of bed nets and IPTp-sulfadoxine-pyrimethamine (SP) which has not been evaluated in America.

Particularly in Colombia, to avoid the complications and the sequelae of PAM, new cases are actively detected using thick blood smears during prenatal control in endemic areas [19]. However, most pregnant women at risk live in remote rural endemic areas, where reagent stability issues, limited access to microscopes, unstable electricity supply, and lack of experienced personnel limit diagnosis and appropriate treatment of the disease [20, 21]. Additionally, there is a high prevalence of sub-microscopic infections associated with intrauterine growth restriction, maternal anaemia, LBW, and continual transmission [22–24].

Molecular testing has shown the best results in terms of PAM diagnosis, including sub-microscopic infections; however, its high costs, lack of infrastructure, equipment and trained personnel, make it unfeasible in endemic areas. Conversely, the detection of *Plasmodium* spp. antigens through lateral flow immunochromatography devices, known as rapid tests, can be applied in the field, they do not require specialized personnel or equipment, they are easy to use, and have showed an adequate diagnostic performance when compared to PCR [25], even superior to microscopic tests [20]. Certain studies support this evidence, reporting the cost-effectiveness of rapid testing, that has represented cost-savings for the health system in some scenarios [26]. These tests have demonstrated to be an alternative for diagnostics in areas where microscopic tests are not available, ensuring timely diagnosis and treatment of pregnant women and preventing negative effects for them and their children, as well as optimizing the use of resources in the health sector.

In Colombia, the cost-effectiveness of these tests is unknown. This study was conducted to estimate the cost-effectiveness of rapid tests type III (*Plasmodium falciparum*/*Plasmodium* spp P.f/pan) in the diagnosis of gestational malaria, compared to microscopic tests, from an institutional perspective in Colombia.

## Methods

### Type of study

Cost-effectiveness of gestational malaria diagnosis from an institutional perspective using a decision tree.

### Population, intervention, comparator, outcome, time, and resources (PICOT-R)

Population: 3,000 pregnant women with malaria. Since the number of cases of gestational malaria was unknown, the figure was calculated using the following data: 163,000 births per year (as a proxy for the number of pregnant women), 3.5 million inhabitants from 241 endemic rural municipalities [27] without malaria control (approximately 7.3% of the Colombian population). The maternal population obtained, of approximately 12,000 women exposed to malaria, with an estimated prevalence of 25%, would result in 3,000 pregnant women affected.

### Intervention

Qualitative detection of *Plasmodium* spp antigens. Histidine-rich protein II (HRP2) expressed by *P. falciparum* or parasite lactate-dehydrogenase (pLDH) metabolic enzyme, expressed by all species of *Plasmodium* spp. (Pf/pan) using lateral flow immunochromatography devices containing antigen-specific monoclonal antibodies from mice.

After using a cotton swab to clean the fingertip with alcohol, it is pricked on one side using a sterile lancet. The first drop of capillary blood is discarded. Using a capillary tube (5 µL), the whole blood sample is collected until it reaches the black line and transferred to the cassette sample well. Four drops of diluent are added vertically for testing in the well. The result is read after 15–30 min. A result is considered negative if a colour band is observed in line ‘C’; it is *P. falciparum*-positive if 2 colour bands are observed in lines ‘P.f’ and C, or 3 colour bands in lines P.f, ‘Pan’ and C; the test is considered positive for another species of *Plasmodium* (*Plasmodium vivax*, *Plasmodium malariae*, *Plasmodium ovale*) if 2 colour bands are observed in lines Pan and C. A colour band should always be observed in line C, otherwise it is considered as an invalid result and the test must be repeated.

### Comparator

Thick blood smear through conventional microscopic test.

### Outcomes

DALY, a combined measure that considers morbidity, mortality and disability, it was used to calculate and study the burden of the disease. It is equivalent to a year of healthy life lost due to illness, disability or premature death and allows for comparison between different pathologies or interventions, and it helps in decision making related to health and resource prioritization [28, 29].

The following components were taken into account: (i) life expectancy taken from previous studies about the burden of the disease in Colombia; (ii) age weighting, which confers greater importance to the years of healthy life of young people due to their greater reproductive capacity; (iii) disability weighting, which reflects the relation between the specific disability during the disease’s duration and the time lost due to mortality within a range from 0 (zero), for perfect health, to 1 (one) for a health status leading to death; and, (iv) discount rate, which gives greater importance to current benefits compared to future ones [29]. For the estimation, years of life lost (YLL) due to premature death and years lived with disability (YLD) due to a disease with a certain severity and duration are combined.

DALYs were calculated using the WHO Global Burden of Disease study project Excel spreadsheet by introducing the following data from SIVIGILA (National System of Public Health Surveillance, in Spanish) and studies about malaria burden of disease in Colombia [27, 29–31]:

K = 0.5. Age weighting as a modulating factor (0 = No Weight. 1 = Total Weight).

β = 0.04. Parameter for age weighting function.

r = 0.03. Annual discount rates for life years in terms of time preference.

C = 0.1658. Model constant, standard-age weighting.

A: Age of death due to gestational malaria.

L: Life expectancy of 80 years for men and 82.5 for women.

D: Disability weight, 0.195 for cases in women under 15 years of age and 0.172. for women over 15 years of age, according to the WHO’s disability weights.

As: Patient age at the onset of the disease, considering the percentage distribution of malaria cases in women from adolescence to 44 years of age, the average onset age in each group with five-year interval according to the WHO, and studies about the burden of the disease in Colombia.

Ld: Duration of the event according to studies about the burden of disease in Colombia: 10.4, 23.3, and 36.5 for age groups between 5–14, 15–29, and 30–44 years, respectively [27, 29, 31].

### Time horizon

Less than one year, consistent with the duration of pregnancy and prenatal control that include screening for malaria in endemic areas [32].

### Resources

Standard costing was performed and validated by three experts using publications on PAM for the identification, measurement and assessment phases. The results were expressed per patient, in Colombian pesos (COP-2019). In the first phase, the cost-generating events described in the management guides and by the experts were identified, which included costs for sampling (laboratory assistant), diagnostic test execution and results reporting (reagents and bacteriologist), costs of treatment (medication, medical attention) and thick blood smears for follow-up of positive cases. In the measurement phase, the amount and frequency for each identified cost was determined. In the assessment phase, data from the Colombian tariff manuals, such as SOAT (Mandatory Traffic Accident Insurance, in Spanish) and SISMED (Prescription Drug Prices Information System, in Spanish), were considered. Since the cost of rapid tests for malaria diagnosis is not included in tariff manuals, micro-costing was performed according to the prices quoted by test suppliers in Colombia.

### Data sources

The number of occurred cases was calculated using records from the Colombian Health Situation Analysis (ASIS) and reported cases at endemic areas in SIVIGILA. The probabilities of true positives and false negatives were obtained from a meta-analysis on the performance of Type III (Pf/pan) rapid tests diagnostic performance studies *versus* molecular diagnosis by PCR [25] conducted by Cochrane Collaboration. Also, a diagnostic evaluation study on the performance of thick blood smears through conventional microscopy and automated microscopy *versus* molecular diagnosis by PCR [33] was considered. The probability of receiving treatment or not was calculated based on the prenatal control service in rural Colombia, as reported in the National Demographic and Health Survey (ENDS, in Spanish; 2015) [32].

### Description of the decision model

**Fig. 1:**
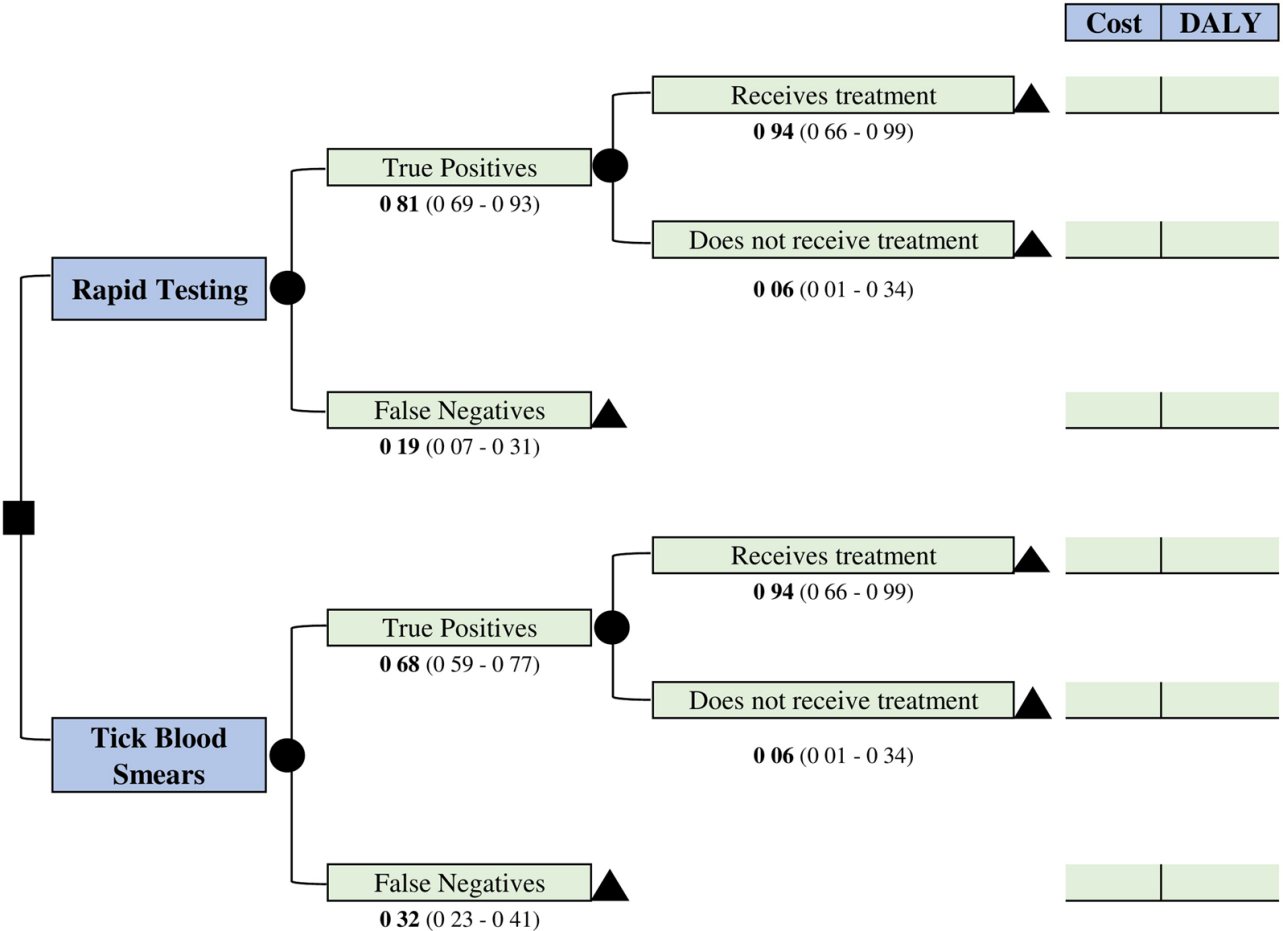
Decision model to determine the cost-effectiveness of gestational malaria diagnosis using rapid tests

Decision tree whose initial node shows both the alternatives compared. The stages evaluated correspond to the classification of cases as true positives or false negatives; in case of a positive diagnosis, the second stage includes receiving or not receiving treatment (Fig. 1).

### Assumptions of the model

Diagnostic test specificity shows a trend to 100% (the probability of false positives tends to zero) [25, 33]. When the reference test is PCR, sub-microscopic infections are determined according to the proportion of false negatives [25]. The efficacy of anti-malarial treatment shows a trend to 100% (treatment failure tends to zero) [34]. In case of failure, the second treatment is applied as part of the prenatal control programme.

### Cost-effectiveness analysis

The average cost-effectiveness ratio was estimated as the cost of each DALY per type of diagnostic test, and incremental cost-effectiveness as the cost of each additional DALY after diagnosis with rapid tests compared to thick blood smears.

$$ Incremental\;cost - effectiveness\;ratio\left( {ICER} \right) = \frac{C}{D}\frac{{Cost\;of\;rapidtests - Cost\;of\;thick\;blood\;smears}}{{Rapidtests\;,\;DALY - Thick\;blood\;smears\;,\;DALY}} $$

### Sensitivity analysis

The uncertainty of the decision model components was assessed through probabilistic sensitivity analysis with Monte Carlo simulations, including a microsimulation with 1,000 individuals, an acceptability curve for different thresholds or willingness to pay, and net monetary benefit (NMB) and expected value of perfect information (EVPI). Thresholds according to WHO [35] were included: values < 1 per capita GDP (approximately US$ 6,000 for Colombia) are indicative of a highly cost-effective, and values < 3 per capita GDP as cost-effective. Regarding probabilities, a β (beta) distribution takes values within a limited range between 0 and 1; regarding DALYs and costs estimates, a γ (gamma) distribution that could take any value greater than 0 was used. The limits of the confidence intervals for diagnostic sensitivity (true positives and false negatives) of the meta-analysis [25] and the diagnostic evaluation study [33] were taken as measures of variability. For costs, a dispersion between 10 and 50%, corresponding to the variability of the tariffs set for health services contracting in Colombia, was considered, so that the results were consistent to the actual dynamics of payment and contracting in Colombia. DALYs were estimated with a relative variation of 20%.

## Results

Regarding rapid tests, a micro-costing methodology was used to assess 25 tests with an average cost of 116,400 COP, which could vary by 22% if additional payment is made for the *P. falciparum/P. vivax*-specific test. In addition, the cost of a laboratory assistant (2 min), a bacteriologist (20 min) and aseptic management materials were included. Materials supplied by test providers were not considered. The cost of a thick blood smear, as it appears in the SOAT tariff manual, includes the entire procedure. The cost per species only differed in the cost of the drug, only an averaged value was considered given that the difference was close to US$1. The persistence of parasitic antigens limits their use for follow-up testing [25]; therefore, follow-up includes two control thick blood smear tests and one medical consultation.

The total cost of the procedure involving a rapid test was US$41.6 per patient, 6.8% of which was related to the diagnosis. The cost of the procedure using thick blood smears was US$42.4, with 8.6% for test performance. For both strategies, the largest proportion of the total cost was for treatment and follow-up (Table 1).

**Table 1:** Description of the costs of rapid tests and thick blood smears

Identification	Measurement	COP^a^ value	Total in COP (US$)^a^
Diagnosis with rapid test			*9,250 (2.82)*
Diagnostic test	1	4,656	4,656 (1.42)
Other* reagents* (gloves and aseptic management)	1	187	187 (0.06)
Laboratory assistant	1	117	117 (0.03)
Bacteriologist	1	4,290	4,290 (1.31)
Diagnosis with thick blood smear			*11,900 (3.63)*
Test value	1	11,900	11,900 (3.63)
Treatment			*37,317 (11.37)*
Medical attention	1	33,100	33,100 (10.09)
Global medication	1	4,217	4,217 (1.28)
Medication *P. falciparum*	24	242	5,800 (1.77)
Medication *P. vivax*	10	264	2,635 (0.80)
Treatment Follow-up			*90,000 (27.42)*
Follow-up test	2	11,900	23,800 (7.25)
Medical Follow-up	2	33,100	66,200 (20.17)

^a^1 American Dollar (US$) = 3,282 Colombian Pesos (COP)

Average cost-effectiveness (ACE) of diagnosis and treatment with rapid tests was US$56.6, and with thick blood smear, US$49.7, per DALY. The incremental cost-effectiveness ratio (ICER) showed that each additional DALY, when diagnosis is obtained using rapid tests, equals US$101.2, compared to thick drop smear. High variability was observed in cost estimation for rapid tests, with a standard deviation of US$4,293 and a range between US$412 and US$95,039. For thick blood smear tests, standard deviation was US$3,818 with a range between US$443 and US$81,362. Likewise, standard deviation for effects was 546 DALYs with a range between 98 and 1,536 for rapid tests and 356 DALYs for thick blood smear tests, with a range between 82 and 1,290. Despite this variability, the sensitivity analysis showed that most simulated cases were in quadrants I and III of the incremental cost-effectiveness plane, demonstrating that rapid tests were highly cost-effective in 70% of cases, with a cost below US$1,200. Only 5% of cases showed dominance of rapid tests compared to thick blood smears (Table 2).

**Table 2:** Estimation of the average and incremental cost-effectiveness ratio and stochastic analysis of its sensitivity

Cost-effectiveness analysis		
Strategy	Costs (US$)/effectiveness (DALY)	Cost-effectivenes (US$)
Rapid tests	66,936 / 1,182	56.6
Thick blood smears	50,838 / 1,023	49.7
Incremental analysis	16,098 / 159	101.2

Sensitivity analysis		
Rapid test	Cost (US$)	Effectiveness
Mean ± Deviation	66,936 ± 4,293	1,182 ± 546
Median	95,039	1,536
Range	412–95,039	98–1,536
Thick blood smears		
Mean ± Deviation	50,838 ± 3,818	1,023 ± 356
Median	81,362	1,290
Range	443–81,362	82–1,290

1000 simulations		
Quadrant	Incremental cost-effectiveness	Frequency (%)
I (IE > 0 IC > 0)	ICER < US$ 1,200	700 (70)
I (IE > 0 IC > 0)	ICER > US$ 1,200	0 (0)
II (IE < 0 IC > 0)	Lower	0 (0)
III (IE < 0 IC < 0)	ICER < US$ 1,200	250 (25)
III (IE < 0 IC < 0)	ICER > US$ 1,200	0 (0)
IV (IE > 0 IC < 0)	Higher	50 (5)

**Fig. 2:**
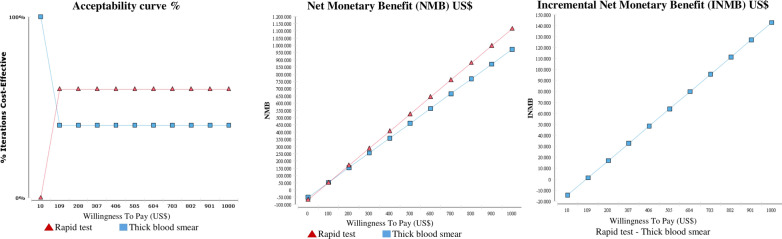
Cost-effectiveness acceptability curve and net monetary benefit (million dollars) of rapid test and thick blood smears according to different payers’ willingness to pay

As shown in the acceptability curve, the probabilistic sensitivity analysis evidenced that, when willingness to pay is above US$100, rapid tests are more cost-effective than thick blood smears. Also, facing the selection of an optimal strategy, and with a willingness to pay of US$200, 60% of the simulated cases show that rapid tests are more cost-effective. The NMB estimation for different payers’ willingness to pay (WTP) also shows that, when WTP equals US$100, rapid tests are the strategy with the highest monetary benefit: in incremental terms, rapid tests sum up to US$10,000 when WTP is equivalent to $200, and up to $150,000 when WTP is equivalent to $1,000, respectively (Fig. 2).

With a WTP of US$18,000, EVPI (incremental NMB) was equal to US$2.5 million; with a WTP of US$12,000, it was US$1.7 million; with an WTP of US$6,000, EVPI was equal to US$822,929; with a WTP of US$500, EVPI was equal to US$28,813 and, with a WTP of US$200, EVPI was equal to US$11,281. This implies that the cost of uncertainty (maximum amount payable that the system is willing to pay in order to implement rapid tests) is relatively low.

## Discussion

This is the first comprehensive economic assessment in Colombia with a cost-effectiveness analysis of gestational malaria diagnosis and treatment at endemic areas. This study collected high-quality scientific evidence that may be used by the government in prioritizing financing strategies for this type of intervention, in order to avoid the negative outcomes of malaria for mother and child. This intervention would also provide benefits for the rest of the population, by interrupting the transmission of the parasite. This would help in advancing towards disease elimination and in the development of other strategies included in the Regional Malaria Elimination Initiative (IREM, in Spanish) [36] by extrapolating the results obtained to contexts under similar conditions.

Even when there have been advances in malaria control over the Americas, the disease remains as a major public health issue in many countries of the region [27]. Particularly, according to various studies conducted in Colombia, the prevalence of the infection in pregnant women is variable and depends on the type of diagnostic test, with values ranging from 9.1% for microscopic tests [37] to 49% with PCR [22]. In addition, it has been reported that up to 45% of pregnant women are asymptomatic and 37% have sub-microscopic infections; this contributes to sub-diagnosis, epidemiological surveillance problems, delayed treatment and an increased risk of complications [22, 38]. In this regard, the WHO argues that active diagnosis is the key to malaria elimination in medium and low-endemic countries by allowing for species-specific treatment, infections detection and epidemiological map creation for malaria [1, 39].

Microscopy examinations are the most common tool for diagnosis and, although they help to quantify parasitic burden, limitations in equipment and experienced personnel make their implementation impossible in remote areas. Therefore, rapid diagnostic tests become a viable alternative in these areas for the active detection and timely treatment of gestational malaria [39] since they are easy to use and do not require specially trained personnel to be applied. This research confirms that the implementation of this type of intervention is highly cost-effective.

While effectiveness measurement is highly heterogeneous in economic assessments, the results from this study are consistent with others that have showed evidence in favour of the use of rapid tests for the diagnosis of gestational malaria. In 2014, a cost-effectiveness assessment of rapid tests compared to thick blood smears in Africa found that the use of rapid tests would render economic savings for the health system that sum up to US$63.47 per case of gestational malaria. This study also states that rapid tests perform better when diagnosing asymptomatic and sub-microscopic infections [40]. Similar studies in Africa have also concluded that this test renders better results during prenatal screening in areas where microscopic examinations by experts are not available [26, 41].

High rates of *P. falciparum* resistance to sulfadoxine-pyrimethamine in Africa have motivated researchers to seek for alternatives in order to prevent PAM complications. One of the most evaluated is intermittent screening and treatment with artemether-lumefantrine (ISTp-AL), a strategy comparable to the one evaluated in this cost-effectiveness analysis, focused on timely diagnosis and treatment of all pregnant women in risk of contracting malaria by using rapid tests. Studies have shown that, although this type of intervention cannot replace IPTp-SP in highly endemic African countries, its effectiveness is comparable if used in areas with extremely high parasitic resistance or low-endemic regions [42, 43]. A systematic review of economic assessments showed that IPTp schemes were the most effective strategy to lower costs for institutions when compared to PAM management on case-by-case basis. ISTp-AL resulted in an average cost-effectiveness of US$19 per each prevented DALY and ICER equivalent to $130.5. These strategies have proven to be highly cost-effective given that their costs are < 1 the per capita GDP in the countries analysed. This shows that in non-African countries, active screening and specific treatment using rapid tests provide comparable results to those of IPTp [18] in terms of effectiveness.

Although scientific literature worldwide provides extensive evidence of the complications for mother and foetus associated with *P. falciparum*, a predominant species in African countries [44, 45], little information is available about the impact of *P. vivax* on the health of mother and child. Nevertheless, no differences have been reported on inflammation profiles of placental infection, suggesting that the pathogenicity and health outcomes generated by both species are similar [24], with cases of maternal anaemia, LBW and congenital malaria [46–49].

Cost-effective analyses conducted in countries where both species are found, have reported that rapid diagnostic tests show a higher cost-effectiveness rate compared to microscopic examinations or clinical diagnosis. In Afghanistan, rapid tests had higher ICER values than thick blood smears, accounting for US$4.5 per adequately treated patient, compared to clinical diagnosis [50]. In Ethiopia, a region with unstable and seasonal malaria transmission, the ACE of rapid tests was US$1.69 per adequately treated patient and they provided the best results in terms of clinical diagnosis [51].

In Peru, a cost-effectiveness assessment of the general population [52] reported that timely diagnosis and treatment using rapid tests would render savings for the Ministry of Health that sum up to US$190.8 and US$31.4 per additional case of falciparum and vivax malaria, respectively, compared to sending the samples to the nearest laboratory. A similar economic assessment carried out in Brazil reported that microscopic examinations were more expensive and less effective than rapid diagnostic tests. Only if sensitivity was > 90% for *P. vivax* and > 83% for *P. falciparum*, microscopic examinations would be more cost-effective [53].

In Colombia, rural malaria areas show similar conditions to those in Peru, Brazil and other endemic countries. In these areas, additionally to living far from health centres, pregnant women have unmet basic needs and unfavourable social conditions. The application of rapid diagnostic tests would contribute to improving their health conditions by reducing the time to diagnosis, and increasing the number of patients with an adequate diagnosis and timely treatment in places where no equipment, trained personnel or infrastructure are available for diagnosis through microscopic examination; thus preventing the negative effects on maternal–fetal health and stopping the transmission of the parasite [21, 52, 54].

Currently, many types of malaria rapid diagnostic test are available, which differ in their diagnostic performance [55]. In this economic assessment, costing was based on type 3 tests incorporating *P. falciparum*-specific antigens and a genus antigen that detects *Plasmodium* spp (P.f/pan), since this type of tests has shown better diagnostic performance compared to immunochromatography incorporating *P. falciparum* and *P. vivax* (P.f/P.v)-specific antigens [25, 55]. Also, a diagnostic evaluation conducted in Colombia showed poor performance in P.f/P.v tests for the diagnosis of PAM as evaluated in peripheral blood, as well in placenta and the umbilical cord [56]. In addition, the presence of *P. falciparum* with HRP2 protein polymorphism increases the risk of false negatives when using rapid diagnostic tests based on this antigen. Nevertheless, tests incorporating genus-specific antibodies have been reported to diagnose *P. falciparum* infections that would not be detected by species-specific antibodies [39].

Rapid diagnostic tests, as well as microscopic examinations, have detection limits that restrict the diagnosis of sub-microscopic infections. Therefore, in this economic assessment, these infections were considered within the ‘false-negative’ group, to avoid an overestimation of this intervention’s effectiveness and to calculate the burden of disease for pregnant women who have not been correctly diagnosed. Sub-microscopic infections are associated with an increased risk of maternal anaemia and LBW, and are responsible for a high percentage of the transmission in low-endemic areas. They constitute a challenge for malaria elimination schemes because they reduce the performance of diagnostic tests, and consequently the effectiveness of interventions [54, 57]. Despite this limitation, it has been proposed that the persistence of parasite antigens in peripheral blood, which can be detected with rapid tests, would allow for the diagnosis of *P. falciparum* infections during periods of parasite sequestration and *P. vivax* latent infection. They may go undetected when using microscopic examinations and PCR, as malaria parasites do not circulate in peripheral blood. This would help in providing timely treatment and reduce false negatives, which may increase the risk of complications [39].

An additional parameter is the efficacy of current treatment against *P. falciparum* and *P. vivax* for pregnant women. In Colombia, an efficacy study reported that the treatment scheme against both species was 100% effective, with mild adverse effects, comparable to those reported among non-pregnant population [34]. Consistently, the intervention, with a correct diagnosis and timely treatment, is expected to achieve a full cure rate among the pregnant population, which is why the likelihood of anti-malarial resistance was not considered in this economic assessment. However, the costing of follow-up medical attention for treated pregnant women was included, to help in the identification of patients with treatment failure who would need second treatment during prenatal visits. It should be noted that in African countries where the main control strategy is IPTp-SP, the intervention continues to be cost-effective even in areas of parasite resistance, as the burden of disease is reduced by avoiding the negative effects on pregnant population without resistance and breaking the chain of infection of the parasite [42, 43, 58]. A similar situation could be achieved in Colombia by using rapid tests to expand the coverage of diagnosis and treatment.

This study has shown evidence to demonstrate the high cost-effectiveness of rapid tests for the specific diagnosis and treatment of malaria during pregnancy. The consistency of the results evaluated during the sensitivity analysis, show that even with a cost-effectiveness < 5 times the per capita GDP, this is a high value for most scenarios, compared to thick blood smears. Although few studies evaluate malaria intervention in pregnant women, the results of this study are comparable to those from other countries [18, 40, 50–53], even including estimates from general populations sharing epidemiological and sociodemographic characteristics similar the ones in Colombia.

Limitations of this study include the lack of standardization for some costs of rapid tests and the exclusion of effects such as reduced diagnosis and treatment time, DALYs for maternal anaemia, severe malaria, abortion, congenital malaria, LBW, and stillbirth in the evaluation [35]. In addition, malaria affects rural areas with high social inequity, generating additional expenditures for families associated with loss of productivity due to hospitalization or medical consultations for the management of maternal or fetal complications [59]. A subsequent evaluation from a social perspective would be desirable, including additional outcomes that would provide greater evidence of the intervention’s cost-effectiveness.

## Conclusion

The use of rapid tests for the timely diagnosis and treatment of gestational malaria is a highly cost-effective strategy in Colombia. The Colombian health system would have to invest US$101 for each additional DALY compared to thick blood smears, after incorporating rapid tests during prenatal control. These results are consistent with the results of publications about countries where rapid diagnostic tests have shown high cost-effectiveness, even rendering savings for health systems. They also have demonstrated that they are effective not only for pregnant women, but also for the general population. This evidence shows that active diagnosis and timely treatment through cost-effective tests available in remote locations constitute a key intervention to prevent uncomplicated cases from progressing to serious illness or causing negative effects on maternal health and the fetus, and contributing to the attainment of the elimination target set by IREM and other WHO initiatives.

## Data Availability

The data used and/or analyzed during the current study are available under reasonable request to the author.

## Abbreviations

AL: Artemether-lumefantrine
ACE: Average cost effectiveness
DALY: Disability-adjusted life year
EVPI: Expected value of perfect information
GDP: Gross domestic product
HIV: Human immunodeficiency virus
IPTp-SP: Intermittent preventive treatment in pregnancy with sulfadoxine-pyrimethamine
ICER: Incremental cost-effectiveness ratio
IREM: Regional malaria elimination initiative (*Iniciativa Regional de Eliminación de la Malaria*)
ISTp-AL: Intermittent screening and treatment in pregnancy with artemether-lumefantrine
LBW: Low birth weight
NMB: Net monetary profit
P.f/P.pan: *Plasmodium falciparum*/*Plasmodium* Spp.
P.f/P.v: *Plasmodium falciparum*/*Plasmodium vivax*
PAM: Pregnancy-associated malaria
PCR: Polymerase chain reaction
WHO: World Health Organization
WTP: Willingness to pay
YLL: Years of life lost
