# Correction to: Cost-effectiveness of rapid diagnostic tests, compared to microscopic tests, for the diagnosis and treatment of gestational malaria in Colombia from an institutional perspective

**DOI:** 10.1186/s12936-020-03525-w

**Published:** 2021-01-20

**Authors:** Deisy Cristina Restrepo-Posada, Jaime Carmona-Fonseca, Jaiberth Antonio Cardona-Arias

**Affiliations:** 1 University of Antioquia, Medellin, Colombia; 2 Department of Medicine, University of Antioquia, Medellin, Colombia; 3 School of Microbiology, University of Antioquia, Calle 70 Number 52–51, Block 5, ofce 103, Medellin, Colombia

## Correction to: Malar J (2020) 19:400 10.1186/s12936-020-03472-6

Following publication of the original article [1], the authors reported that the following grant details had been omitted from the Funding declaration of their article: ‘Grant support Project 111577757447. Contract 755-2017./Colciencias’.

The published article has now been corrected to include these details in the Funding declaration.

The authors apologize for any inconvenience caused.
